# Study on the dynamic adsorption and recycling of phosphorus by Fe–Mn oxide/mulberry branch biochar composite adsorbent

**DOI:** 10.1038/s41598-024-51416-w

**Published:** 2024-01-12

**Authors:** Liang Meina, Mushi Qiao, Qing Zhang, Shuiping Xu, Dunqiu Wang

**Affiliations:** 1 School of Envormental Science and Engneering, Guilin Unversity of Technology, Guilin, 541004 People’s Republic of China; 2 Collaborative Innovation Center for Water Pollution Control and Water Safety Guarantee in Karst Area, Guilin, 541004 People’s Republic of China

**Keywords:** Ecology, Environmental sciences

## Abstract

In this study, the Fe–Mn oxide/mulberry stem biochar composite adsorbent (FM-MBC) was prepared and fully characterized by SEM-EDS, XRD, BET, and XPS. The solution pH (3.0, 4.5, and 6.0), initial concentration of phosphorus (10, 20, and 30 mg L^−1^), adsorbent bed height (2, 3, and 4 cm), and solution flow rate (1, 2, and 3 mL min^−1^) were investigated to analyze the breakthrough curves. The results showed that the breakthrough time was shortened as the initial phosphorus concentration, the flow rate increased and the bed height decreased. Higher initial phosphorus concentrations, flow rates, and lower bed heights, led to a faster breakthrough of phosphate ions in the FM-MBC adsorbent. Additionally, it was observed that increasing the pH value was not conducive to the adsorption of phosphorus by the FM-MBC adsorbent. Dynamic adsorption data were fitted to four models (Yoon-Nelson, Thomas, Adams-Bohart, and Bed Depth Service Time), and the R^2^ values of the Thomas and Yoon-Nelson models exhibited minimal variation, suggesting that the dynamic adsorption process of FM-MBC was rather intricate. The saturated fixed-bed column (including FM-MBC) was regenerated with NaOH or HCl, and it was found that a 0.1 mol L^−1^ NaOH solution had the best regeneration effect. XRD analysis showed that the reaction product between the FM-MBC composite and phosphate anions was Fe_3_(PO_4_)_2_·H_2_O. Moreover, the experimental results that FM-MBC can successfully be used to remove phosphorus from actual wastewater.

## Introduction

Phosphorus is a vital element that supports life in the earth’s system and plays a fundamental role in the growth of plants and animals^1^. However, excessive phosphorus can lead to eutrophication in water bodies. This phenomenon triggers the rapid proliferation of algae and other planktonic organisms, resulting in foul-smelling and odorous water, reduced transparency, decreased dissolved oxygen concentration, deteriorated water quality, and even mass mortality of fish and other organisms. Furthermore, the excessive consumption of high-quality commercial phosphorus mines has created an urgent need to develop new methods that can effectively remove wastewater phosphorus pollution and recover phosphorus^2^.

In recent years, various methods have been employed to treat phosphorus-containing wastewater. These methods include chemical precipitation^3^, membrane separation^4^, ion exchange^5^, biological processes^6^, photocatalysis^7^, constructed wetlands^8^, and adsorption^9^. Among these techniques, ion exchange, ultrafiltration, membrane separation, and reverse osmosis are adequate for phosphorus removal. However, they have high operating costs and require significant financial investment and operational expenses^10^. Notably, adsorption is a widely used method for treating phosphorus-containing wastewater. It offers several advantages, including excellent adsorption performance, recyclability, low cost, simplicity of operation, and the ability to remove and recover target substances through adsorption–desorption processes^11–13^.

Over 6 million tons of mulberry branches are harvested annually in the Guangxi region. Unfortunately, these branches are often burned, causing atmospheric pollution, resource wastage and environmental harm^14^. It is imperative to find an economical utilization of this abundant agro-waste. However, the adsorption and removal of pollutants by pristine mulberry branch biochar is relatively poor, so developing modified biochar with higher adsorption capacity has always been people’s goal.

Iron and manganese oxides possess unique surface activity, allowing them to adsorb and degrade various inorganic and organic environmental pollutants^15^. Iron and manganese oxides tend to aggregate, which poses challenges for their practical applications. To overcome this limitation, researchers have recently focused on combining iron and manganese oxides with other adsorbents, such as bio-apatite-based material^13^, biochar and chitosan to create composite adsorbents. This approach not only enhances their adsorption performance through synergistic effects but also resolves the issue of oxide aggregation.

The study indicates that previous studies have primarily focused on the static adsorption of phosphorus using pristine biochar and modified biochar^16,17^. Dynamic adsorption is often preferred for removing contaminants from industrial effluents due to its operational simplicity, high pollutant removal efficiency, and ease of scale-up from laboratory processes. The choice of material for the packed column is crucial in dynamic adsorption. Importantly, obtaining a reliable prediction of the breakthrough curve under specified operating conditions can effectively design and operate the packing adsorption process. However, a limited number of reports are available concerning the utilization of a composite material consisting of Fe–Mn oxides and mulberry branch biochar (FM-MBC) for investigating the dynamic adsorption behavior of phosphorus.

This study used mulberry branches as the pristine material to prepare FM-MBC. The dynamic adsorption experiments investigated the impact of solution pH, initial phosphorus concentration, bed height, and flow rate on the adsorption breakthrough curve. To predict the breakthrough curve, the experimental data obtained from the dynamic adsorption experiments were fitted with four different models: Adams-Bohart, Thomas, Yoon Nelson, and BDST. These models were used to evaluate the parameters of the fixed bed and provide insights into the adsorption process. Finally, a combination of dynamic adsorption experiments and characterization results was used to investigate the potential mechanisms of phosphate elimination by the FM-MBC composite.

## Materials and methods

### Preparation and characterization of FM-MBC

The method for preparing the FM-MBC is based on our previous works^18^. Various analytical techniques were employed to characterize the structure of the FM-MBC material, including SEM with Energy Dispersive X-ray Spectroscopy (SEM-EDS), Brunauer–Emmett–Teller (BET) surface area and pore size analysis, X-ray Diffraction (XRD), and X-ray Photoelectron Spectroscopy (XPS).

### Column adsorption experiment

**Figure 1:**
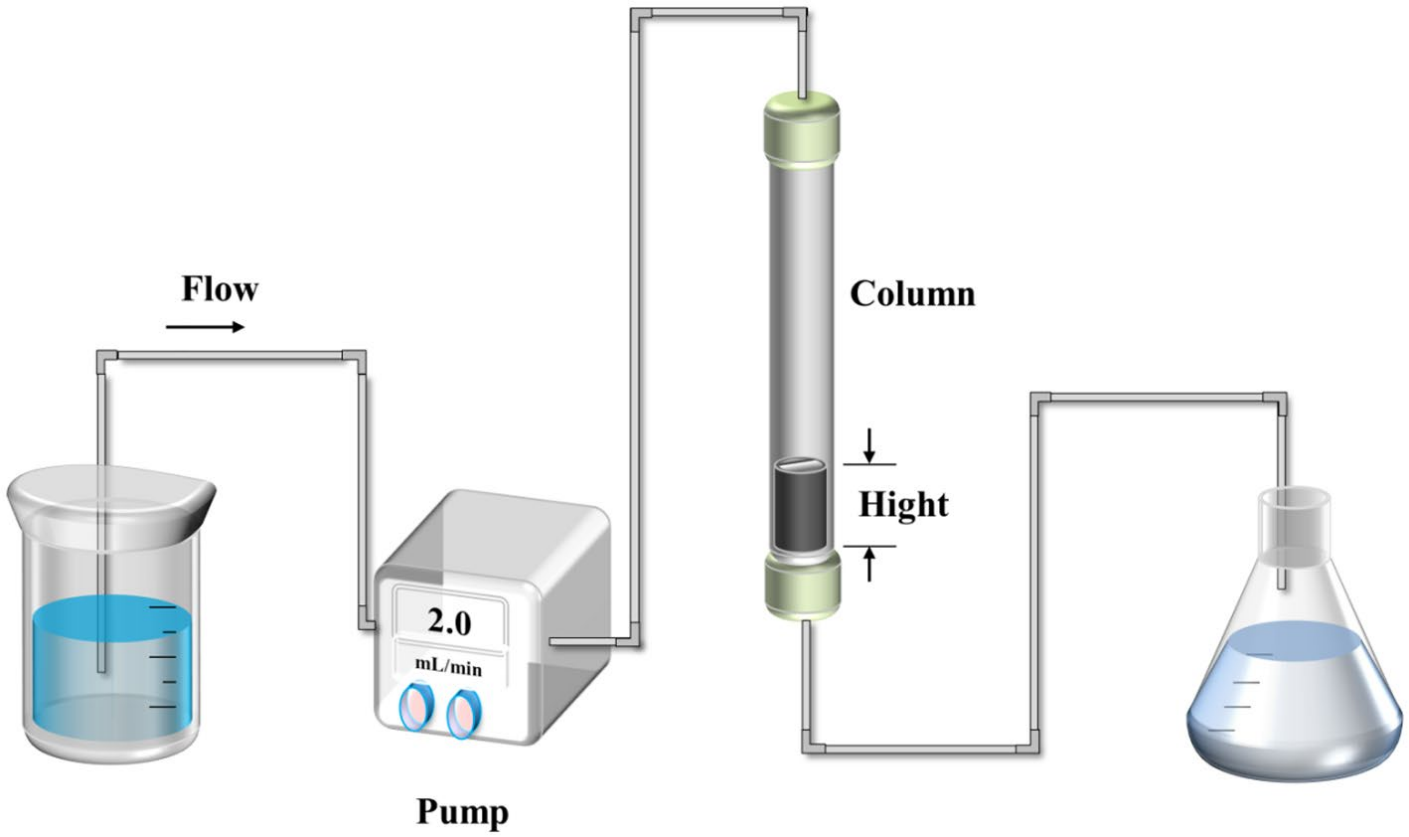
Dynamic adsorption experiment equipment.

A glass column with a diameter of 1 cm and a length of 15 cm was chosen as the adsorption column (Fig. 1). The dynamic experimental setup is shown in the diagram (Fig. 1). The chromatographic column was filled with 1.0 ~ 2.0 g of FM-MBC material, and the nuts at both ends were tightened. The pipeline was connected according to the illustration. Following this, the peristaltic pump was activated, and the column was flushed with ultrapure water for 3 ~ 5 min to eliminate any bubbles present. Afterward, the pump was stopped. To conduct the dynamic adsorption experiments, the inlet pipe was placed into the phosphate solution. The experimental conditions involved variations in the bed height (1.0, 1.5, and 2.0 g FM-MBC), flow rate (1 ~ 3 mL min^−1^), and initial phosphorus concentration (10 ~ 30 mg L^−1^). The dynamic adsorption experiments were performed at a temperature of 25 °C. At specific time intervals (5, 10, 15, 30, 45, 60, 75, 90, 105, 120, 135, 150, 165, 180, 210, 240, 270, 300, 360, 420, 480, 540, 600, 720, 840, 960, 1080, 1200, 1320, and 1440 min), 10 mL samples were collected, ensuring equal sample volumes each time.

### Determination of phosphorus concentration

The concentration of total phosphorus in solution was measured by the ammonium molybdate spectrophotometric method.

### Breakthrough curve performance analysis

The breakthrough curve represents the adsorption performance of a fixed bed. In general engineering, the breakthrough point is defined as the moment when the mass concentration of the target solution in the effluent reaches 2–5% of the mass concentration of the influent. On the other hand, the adsorption saturation point is reached when the mass concentration of the solution in the effluent comes to 95–98% of the mass concentration of the influent^19^. In this study, the breakthrough point is determined by selecting the time point at which the mass concentration of the solution in the effluent reaches 5% of the influent concentration (C_t_/C_0_ = 5%)^20,21^. The time taken to get to this point is defined as the breakthrough time. Similarly, the adsorption saturation point is determined by selecting the time point at which the mass concentration of the influent solution reaches 95% of the influent concentration (C_t_/C_0_ = 95%)^19^.

### Regeneration research

The regenerants included 0.1 mol L^−1^ HCl and 0.1 mol L^−1^ NaOH solution. Firstly, when the adsorption reaction reached equilibrium, the saturated biochar was separated from the solution. Subsequently, the regeneration experiment of the saturated biochar was conducted. 0.1 g saturated biochar was added to a conical flask containing 100 mL of regeneration solution. The flask was then placed in a thermostatic shaker and Oscillate in a water bath constant temperature shaker at 25 °C and 180 r min^−1^ for 48 h. Once the regeneration reaction was completed, the regenerated biochar was removed from the regeneration solution and subjected to washing and drying processes for subsequent experiments. The regeneration efficiency (%) was calculated using Eqs. (S1) in the Supplementary Material S1.

## Results and discussions

### Material characterizations

**Figure 2:**
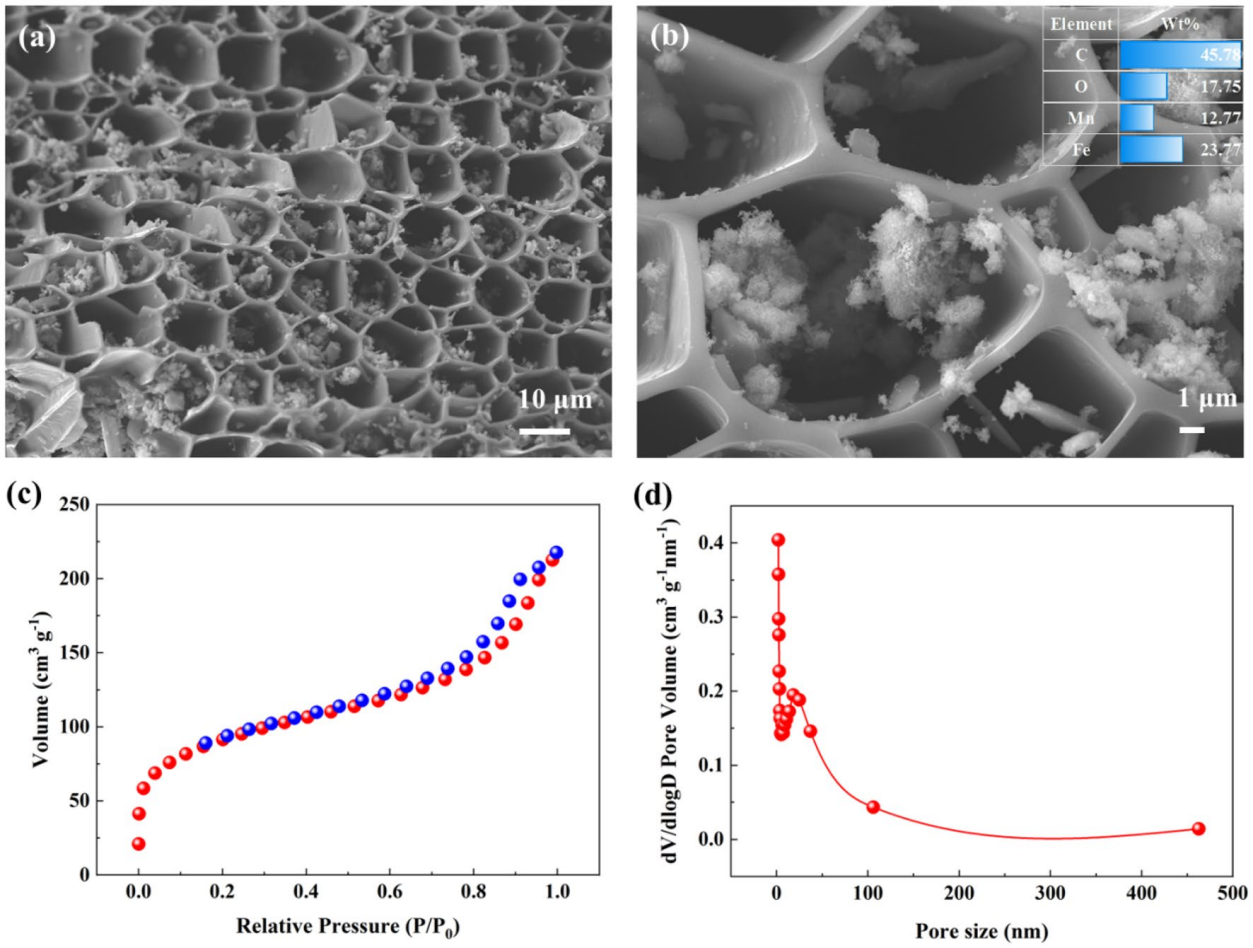
The SEM images at 1000x (**a**) and 5000x (**b**), N_2_ adsorption–desorption isotherms and pore size distribution (**c**, **d**).

The SEM images of the FM-MBC at 1000 × and 5000 × magnification are presented in Fig. 2a, b, respectively. The surface appears flat, and the pore structure exhibits a honeycomb pattern. The internal structure of the pore was observed clearly in Fig. 2b. Upon closer examination, it is evident that there are substances loaded on both the surface and pores of FM-MBC. These substances have been identified as iron and manganese oxides, as confirmed by EDS analysis (Fig. 2b). The properties of FM-MBC are summarized in Table S1. The XPS and XRD of FM-MBC are shown in Fig. S1a, b. Notably, the surface area of FM-MBC is remarkably high 318.53 m^2^ g^−1^, which indicates that FM-MBC is an excellent adsorbent. The pore size distribution of FM-MBC is primarily concentrated in the range of 2 ~ 40 nm (Fig. 2d), with a majority of mesopores and a smaller proportion of micropores and macropores, indicating a typical mixed structure. It is worth noting that the surface area of an adsorbent is a key determinant of its sorption capacity.

### Effects of operating parameters

#### Effect of solution pH

**Figure 3:**
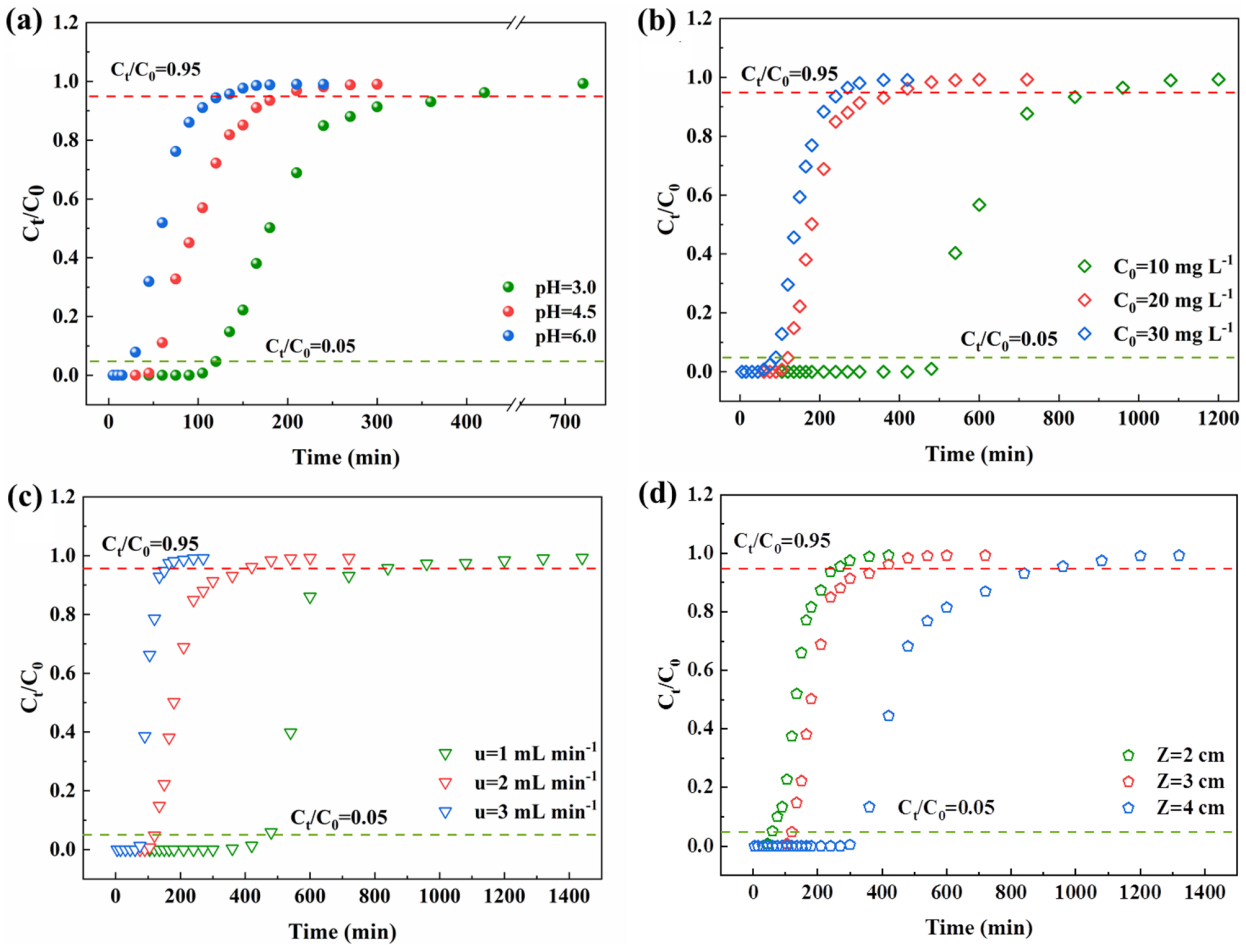
Effect of pH (**a**), initial P concentration (**b**), flow rate (**c**), and bed height (**d**) in the breakthrough curves for the adsorption of phosphorus in FM-MBC.

The solution pH is a crucial parameter in the adsorption process, which influences the physicochemical properties of the interaction between substances and the adsorbent in the solution. The solution pH affects the mechanism by which the solid adsorbent interacts with the concrete surface^22^. The charge properties of the adsorbent surface play a significant role in determining the ionization state of the functional groups on the adsorbent surface. The zero point charge (pH_pzc_) of FM-MBC was measured to be 5.64 (Fig. S2). The pH_pzc_ is the pH at which the adsorbent surface has no net charge. With conditions of pH < pHpzc, the functional groups on the FM-MBC surface are protonated and carry positive charges, which is favorable for the adsorption of the anionic phosphate species (mainly H_2_PO_4_^-^). The effect of pH on the dynamic adsorption of phosphate by FM-MBC is shown in Fig. 3. When the solution pH is 3.0, 4.5, and 6.0, and the breakthrough time (*t*_*b*_) is 124, 53, and 17 min, and then the exhaustion time (*t*_*e*_) is 397, 200, and 126 min (Fig. 3a), respectively. When the pH increases from 3.0 to 6.0, both *t*_*b*_ and *t*_*e*_ of the adsorption column are shorter. This indicates that acidic conditions are favorable for phosphorus adsorption, which is consistent with the batch adsorption experimental results reported by Nguyen^23^. This may be because, in an acidic medium (pH = 3), phosphate species mainly exist in the form of H_2_PO_4_^-^ / HPO_4_^2−^, and H_2_PO_4_^−^ / HPO_4_^2−^ are adsorbed on the FM-MBC surface due to electrostatic interactions with the cationic functional groups^23^. Electrostatic attraction is one of the adsorption mechanisms of FM-MBC for phosphate^23^.

#### Initial phosphorus concentration

It has been reported that the initial phosphorus concentration of the influent also affects the breakthrough curve^24^. The effect of initial phosphorus concentration (10, 20, and 30 mg L^−1^) on the breakthrough curve (C_t_/C_0_) is shown in Fig. 3b. As shown in Fig. 3b, when the initial phosphorus concentration is 10, 20, and 30 mg L^−1^, and *t*_*b*_ is 484, 124, and 90 min, and then *t*_*e*_ is 785, 397, and 255 min, respectively. Within the range of initial phosphorus concentration of 10–30 mg L^−1^, the breakthrough time slightly decreases with increasing phosphorus concentration. When the initial phosphorus concentration of the influent is low, the breakthrough curve is more dispersed and the breakthrough rate is slower. This may be due to the decrease in diffusion coefficient or mass transfer coefficient caused by a lower concentration gradient, resulting in a slower rate of mass transfer. The higher the initial phosphorus concentration of the influent, the steeper the slope of the breakthrough curve and the shorter the breakthrough time^23^. The results indicate that the change in concentration gradient affects the saturation and breakthrough time of the reaction; in other words, the diffusion process is related to the initial solution concentration. With the increase in the initial influent concentration, the loading rate of phosphate increases, and the driving force for mass transfer increases. The adsorption capacity increases with the addition of influent concentration because the high concentration difference provides a more significant driving force for the adsorption process^25^.

#### Effect of flow rate

The breakthrough curves (C_t_/C_0_) of phosphate on FM-MBC at different solution flow rates are shown in Fig. 3c. With the increase in solution flow rate, the slope of the effluent curve increases and the time to reach the breakthrough point and saturation point decreases. When the solution flow rate is 1.0, 2.0, and 3.0 mL min^−1^, and *t*_*b*_ is 469, 124, and 76 min, and then *t*_*e*_ is 808, 397, and 151 min, respectively. This may be due to the difference in solution flow rate leading to changes in Reynolds number. The Reynolds number increases with the increase in solution flow rate. When the Reynolds number is high, the residence time of the adsorbate in the column is insufficient to establish adsorption equilibrium, so the solution leaves the fixed bed before reaching equilibrium, resulting in a shorter breakthrough time. On the other hand, a lower flow rate provides a longer contact time between the phosphate and the adsorbent, resulting in better removal of phosphate in the column^26^.

#### Effect of bed height

The effect of adsorbent bed height on the breakthrough curve of dynamic adsorption of phosphate by FM-MBC is shown in Fig. 3d. The breakthrough curve changes with the variation of bed height, and the change in the breakthrough curve conforms to the S-shaped curve characteristic of an ideal adsorption system. In addition, at a bed height of 2 cm, the effluent concentration increases rapidly, while at a bed height of 4 cm, the S-profile is more pronounced. When the bed height decreases, the axial dispersion phenomenon dominates the mass transfer process, reducing the diffusion of ions, and the solute does not have enough time to diffuse into the entire mass of the adsorbent. With the increase in adsorbent bed height, the adsorption rate of phosphate decreases, the slope of the effluent curve decreases, the contact time between FM-MBC and phosphate increases, and the time to reach the breakthrough point and saturation point is prolonged, thereby increasing the removal efficiency of phosphate in the column. The bed height is 2.0, 3.0, and 4.0 cm, and *t*_*b*_ is 58, 124, and 321 min, and then *t*_*e*_ is 961, 397, and 273 min, respectively. The reason is that the increase in bed height increases the specific surface area and the tortuosity of the fluid channels in the fixed bed, providing more active sites for the adsorption process. The experimental adsorption capacities have been compared with those reported by other authors in similar studies are presented in Table 1.

**Table 1:** Comparison of adsorption capacity of phosphorus for various adsorbent materials.

Adsorbent	Pollutant	q_e_ (mg g^-1^)	References
FM-MBC	Phosphorus	21.11	Present work
MgCl_2_-CeCl_3_ modified wheat straw biochar	Phosphorus	7.74	^27^
Epigallocatechin gallate-iron biochar	Phosphorus	4.61	^28^
Ca-MBCs	Phosphorus	25.60	^29^
Eupatorium adenophorum biochar	Phosphorus	2.32	^30^

### Breakthrough curve modeling

In dynamic adsorption studies for industrial applications, various mathematical models have been developed to describe and analyze the adsorption process. These mathematical models provide a simplified representation of the adsorption behavior and can be used to fit experimental data and predict the breakthrough curve and adsorption capacity^22^. In this study, the Adams-Bohart, Thomas, and Yoon-Nelson models were used to fit the experimental data and predict the breakthrough curve and adsorption capacity.

#### Adams-Bohart model

**Figure 4:**
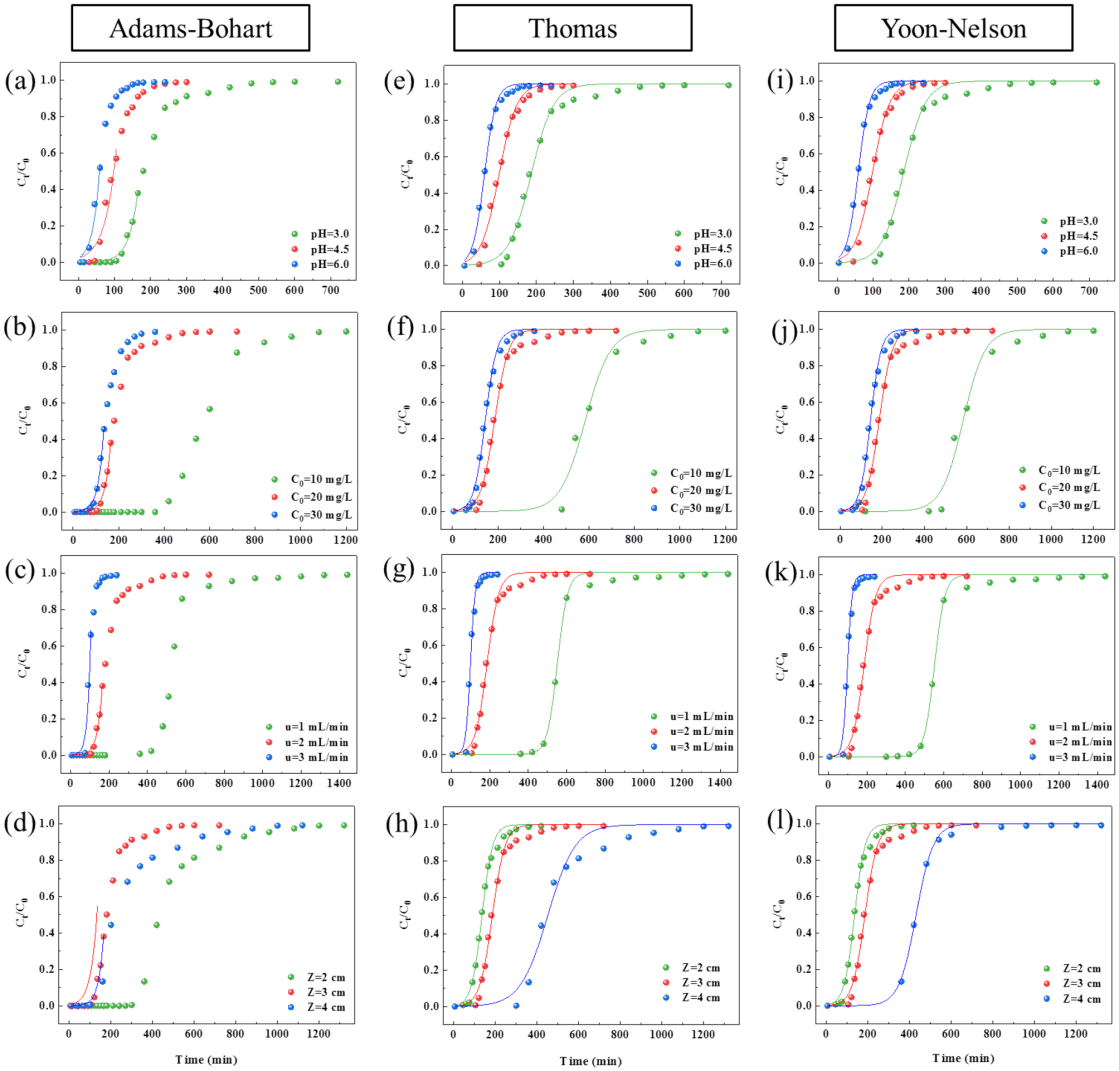
Adams-Bohart, Thomas model and Yoon-Nelson model fitting curves of different solution pH, initial phosphorus concentration, bed height and flow rates (**a**–**d**: Adams-Bohart model, **e**–**h**: Thomas model, **i**–**l**: Yoon-Nelson model).

Adams-Bohart established an equation based on surface reaction theory to describe the relationship between the concentration ratio (*C*_*0*_/*C*_*t*_) and time (*t*) in a continuous system^22^. According to Eq. (S2), nonlinear regression was performed using the sampling time (t) as the x-axis and the natural logarithm of the concentration ratio (*C*_*t*_/*C*_*0*_) as the y-axis (Fig. 4a–d). The Adams-

Bohart model parameters (K_AB_ and N_0_) were obtained at different process variables, which are listed in Table 2. As the flow rate increases from 1.0 to 3.0 mL min^-1^, the K_AB_ increases with the flow rate but decreases with the addition of adsorbent bed height. The range of R^2^ obtained in this study (0.9249–0.9819) is similar to the results reported by the Chen group^22^ and Ramirez et al.^31^. This indicates that in the initial stage of adsorption in the column, the overall system dynamics are primarily controlled by external mass transfer^25^. The correlation coefficient of the nonlinear model was greater than 0.9249 in most cases, which implies an excellent similarity with the experimental data.

**Table 2:** Parameters of Adams-Bohart, Thomas, Yoon-Nelson and BDST model and model for dynamic adsorption of phosphate.

C_0_ (mg/L)	u (mL/min)	Z (cm)	pH	Adams-Bohart model			Thomas model			Yoon-Nelson model		
				K_AB_ × 10^–3^ (mL/mg·min)	N_0_ (mg/mL)	R^2^	K_TH_ × 10^–3^ (mL/mg min)	q_TH_ (mg/g)	R^2^	K_YN_ × 10^–3^ (min^-1^)	τ (min)	R^2^
20	2	3	3.0	2.02	2.51	0.9835	1.65	4.94	0.9892	3.31	185.22	0.9892
20	2	3	4.5	1.61	1.60	0.9249	2.09	2.63	0.9883	4.19	98.50	0.9883
20	2	3	6.0	2.63	0.96	0.9504	3.22	1.57	0.9928	6.54	59.05	0.9928
10	2	3	3.0	2.77	1.71	0.9432	1.81	7.78	0.9892	1.94	582.77	0.9823
30	2	3	3.0	1.40	3.06	0.9818	1.34	5.77	0.9928	4.04	144.14	0.9915
20	1	3	3.0	1.29	2.76	0.9672	4.36	7.35	0.9963	3.71	551.32	0.9971
20	3	3	3.0	1.34	5.65	0.9818	3.95	3.95	0.9819	8.18	98.86	0.9819
20	2	2	3.0	1.66	3.07	0.9819	1.96	5.42	0.9922	3.94	135.54	0.9922
20	2	4	3.0	2.43	5.02	0.9621	0.76	9.02	0.9684	2.42	430.66	0.9999

C_0_ (mg L^-1^): initial concentration; u (mL min^-1^): flow rate; Z (cm): bed height; K_AB_ (mL min^-1^mg^-1^): Adams-Bohart constant rate; N_0_ (mg mL^-1^): saturation concentration of the column; q_TH_ (mg g^-1^): adsorption capacity; K_TH_ (mL mg^-1^ h^-1^): Thomas constant rate; K_YN_ (mL mg^-1^ h^-1^): Yoon-Nelson constant rate.

#### Thomas model

The Thomas model is a widely used dynamic adsorption model for describing breakthrough curves and predicting the adsorption capacity of adsorbents in fixed-bed systems. It assumes no axial diffusion in the adsorption process^32^. According to Eq. (S3), nonlinear regression was performed using the sampling time as the x-axis and the natural logarithm of the concentration ratio *C*_*t*_/*C*_*0*_ as the y-axis (Fig. 4e–h). The parameters (K_TH_ and q_0_) of the nonlinear regression analysis are presented in Table 2. The results show that the fitting effect of the Thomas model (R^2^ range: 0.9684–0.9963) was better than that of the Adams-Bohart model. According to Table 2, as the solution flow rate increases from 1.0 to 3.0 mL min^−1^, the K_TH_ increases with the flow rate, while the q_0_ decreases with the addition in flow rate. The decrease in q_0_ can be attributed to the lower availability of active sites at higher flow rates^31^.

#### Yoon-Nelson model

The Yoon-Nelson model is a classic dynamic adsorption model with a simple expression^31^. However, it is only applicable for describing the adsorption process of simple component adsorption systems. According to Eq. (S4), nonlinear regression was performed with sampling time t as the x-axis and C_t_/C_0_ as the y-axis (Fig. 4i–l). The Yoon-Nelson model parameters obtained by the nonlinear regression are presented in Table 2. As the flow rate increases from 1.0 to 3.0 mL min^−1^, the *K*_*YN*_ decreases with the increase of bed height but increases with the addition of flow rate. The adsorbent material quickly reaches saturation as the flow rate increases^31^. The *τ* decreases with the addition of flow rate and initial phosphate concentration, then increases with the addition of adsorbent bed height. This is because the column reaches saturation faster at a higher initial phosphate concentration and flow rate^32^. The range of *R*^*2*^ is 0.9684–0.9999, which is lower than that of the Thomas model.

#### BDST model analysis

**Figure 5:**
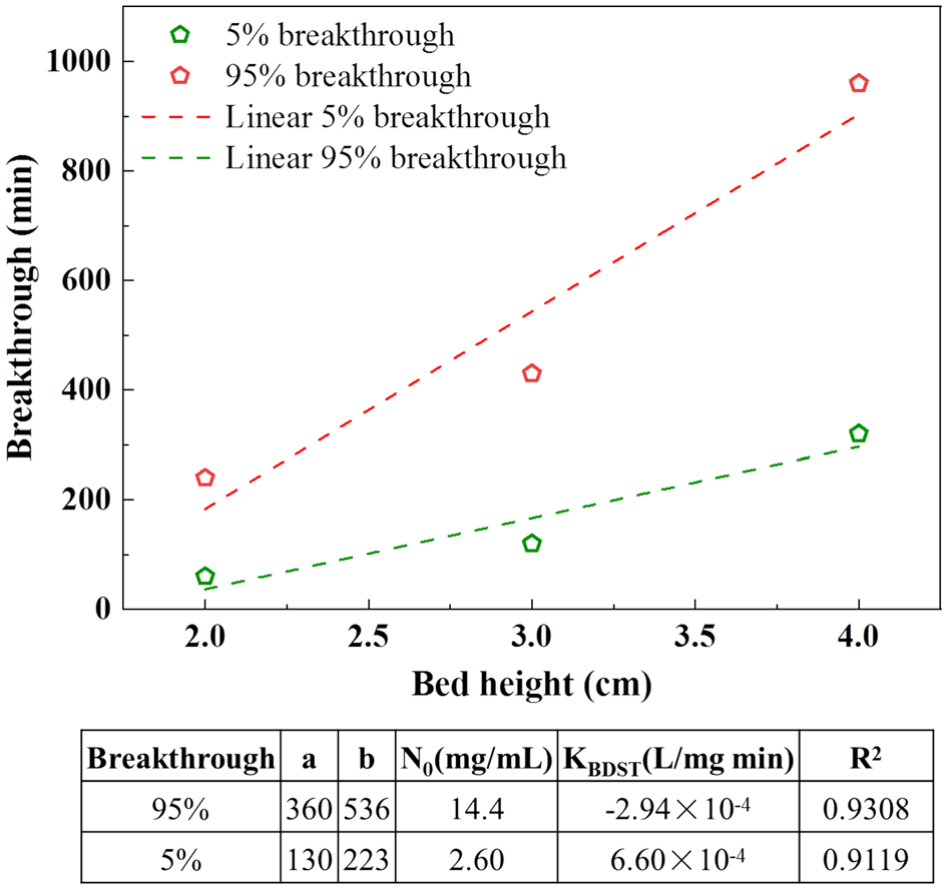
BDST model fitting for C_t_/C_0_ = 0.05 and C_t_/C_0_ = 0.95 saturation at various bed depths.

The relationship between fixed-bed breakthrough time and adsorbent bed height was investigated further in this study (Supplementary Material S4). The variation in the service time of the column was studied for two different values of C_t_/C_0_ (0.05 and 0.95), as shown in Fig. 5. The high correlation coefficients (R^2^ > 0.9119) indicate that the BDST model accurately interprets the breakthrough characteristics of phosphate on the FM-MBC adsorbent. The results of each parameter are summarized in Table S3.

At a flow rate of 2.0 mL min^−1^, the measured breakthrough time had the most average relative error (23.39%) compared to the predicted breakthrough time. Conversely, the flow rate of 2.0 mL min^-1^ had the lowest error (5.83%). This discrepancy can be attributed to the increased contact time between the phosphate and FM-MBC at low flow rates, allowing sufficient time for internal diffusion. However, it should be noted that the BDST model is established based on the assumption of ignoring internal diffusion.

#### Comparative analysis of breakthrough models

Linear and nonlinear fitting methods were employed in the aforementioned Adams-Bohart, Thomas, and Yoon-Nelson models, and their correlation coefficients (0.9249 ≤ R^2^ ≤ 0.9999) were subsequently compared, which showed that nonlinear regression is most appropriate for the analysis of the dynamic adsorption models^−^^22^. The range of R^2^ values obtained for the Thomas model indicates a better fit than the Adams-Bohart model. Moreover, the BDST model also correlated well with the experimental data (Table S3). In contrast, the R^2^ values of the Thomas and Yoon-Nelson models showed slight variation (0.9684 ≤ R^2^ ≤ 0.9963 and 0.9819 ≤ R^2^ ≤ 0.9999), indicating that the dynamic adsorption process of FM-MBC was more complex. Comprehensive comparison, and complete consideration of the breakthrough properties and parameters were necessary, rather than relying solely on one model. Furthermore, the outcomes of the linear fitting method were contrasted with the Supplementary Materials (Fig. S3 and Table S2), along with their corresponding parameters.

### Regeneration performance

The saturated adsorbent was recycled four times using 0.1 mol L^−1^ NaOH and HCl, as shown in Fig. S4. After each desorption, the adsorption capacity of phosphorus decreased to some extent. This decrease can be attributed to the strong binding of some phosphate ions, which penetrate the interior of the adsorbent, making it difficult to achieve 100% desorption efficiency^33^. Specifically, after three consecutive cycles, the adsorption capacity of FM-MBC for phosphate ions decreased from 96.08 to 72.88% (Fig. S4). This decrease may be attributed to attractive sites that cannot be completely reversed during desorption. Specifically, some adsorbed phosphate ions are located deep inside the FM-MBC and exhibit strong adhesion to the adsorbent, decreasing the number of effective active adsorption sites^34^. Although the adsorption capacity of FM-MBC decreases with the increase of recycling times, the phosphorus removal efficiency after four consecutive recoveries is more than 72%, indicating that iron-manganese-modified biochar has high reusability and is a promising phosphorus adsorbent.

### Phosphorus removal from actual wastewater

In this study, we aim to investigate the suitability of the FM-MBC composite for widespread phosphorus adsorption. Actual samples were taken from four different sources, including river water (RW), agricultural wastewater (AW), municipal wastewater (MW), and pharmaceutical wastewater (PW). After filtration, each sample, with initial phosphate concentrations of 10 and 30 mg L^−1^, was treated with 2.0 g of the FM-MBC composite. Subsequently, we assessed the efficacy of phosphate ion removal.

**Figure 6:**
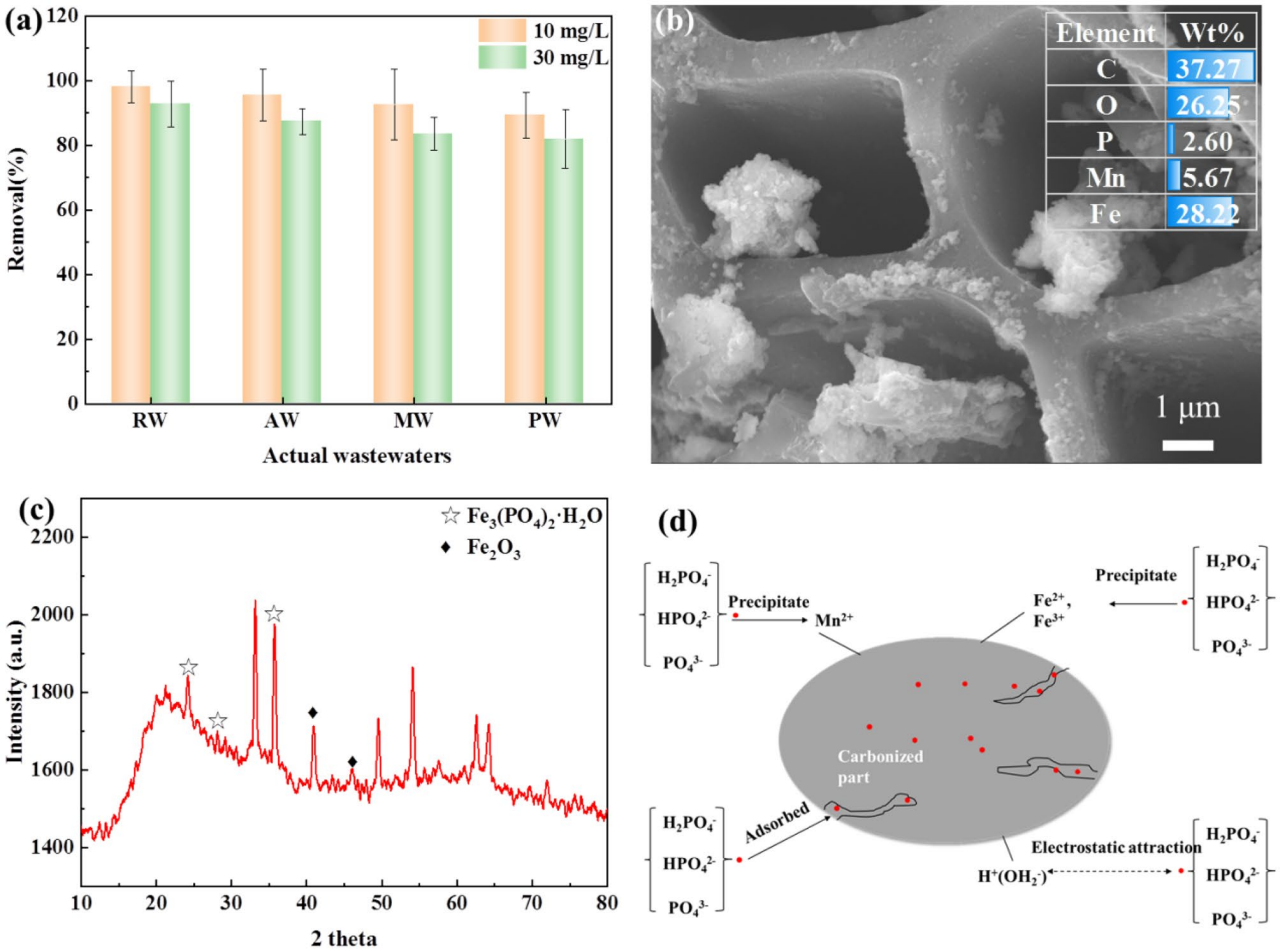
Removal efficiency of phosphorus from actual samples (**a**), phosphorus removal mechanisms by FM-MBC composite (**b**, **c**, **d**). *Note* river water (RW), agricultural wastewater (AW), municipal wastewater (MW), and pharmaceutical wastewater (PW).

The percentage of phosphate removal from the collected samples followed a specific order: river water > agricultural wastewater > municipal wastewater > pharmaceutical wastewater. As depicted in Fig. 6a, the FM-MBC composite showed high adsorption capabilities, removing more than 98.1%, 95.5%, 92.6%, and 89.3% of 10 mg L^−1^ phosphorus after 240 min for samples obtained from river water, agricultural wastewater, municipal wastewater, and pharmaceutical wastewater, respectively. However, when the initial phosphorus concentration increased to 30 mg L^−1^, the percentage of phosphorus removal decreased in the same pattern for all sources. The above results suggest that the FM-MBC composite exhibits promising potential for efficiently removing phosphate from water samples.

### Phosphorus removal mechanism

The EDS spectrum depicted in Fig. 6b illustrates the changes in the elemental composition of FM-MBC following phosphorus adsorption. A comparison with Fig. 2b reveals a significant increase in the oxygen percentage from 7.02 to 22.31% in FM-MBC. Additionally, the ratio of Mn and Fe decreased from 15.57 to 9.88% and from 47.62 to 34.24%, respectively. Notably, the EDS spectrum of FM-MBC did not exhibit any phosphorus peak; While, a distinct peak characteristic of phosphorus is observed after phosphorus adsorption. This confirms the presence of phosphorus on the surface of FM-MBC following the adsorption process. These findings suggest that surface adsorption or ion exchange may account for removing phosphorus from the solution. Under acidic conditions, FM-MBC illustrates effective adsorption of phosphate ions, primarily through electrostatic attraction. The XRD pattern of the FM-MBC composite was obtained after impregnation in a phosphate solution (Fig. 6c). The three peaks observed at 2θ = 25.7°, 29.5°, and 34.1° correspond to Fe_3_(PO_4_)_2_·H_2_O, indicating the formation of iron phosphate precipitation. Although manganese phosphate precipitation could also theoretically form, its content may be low and undetectable by XRD.

It can be seen from the XPS spectrum (Fig. S1a and Table 3) before adsorption that the prominent element peaks are Fe2p (7.19%), Mn2p (3.16%), O1s (26.98%), C1s (60.72%), and no phosphorus is detected. There are more phosphorus peaks on the FM-MBC after phosphorus adsorption, which indicates that a certain amount of phosphorus is adsorbed on the surface of the adsorbent. It can be seen from Table 3 that the surface elements of FM-MBC after phosphorus adsorption have specific changes, which are Fe2p (5.29%), Mn2p (1.3%), O1s (27.31%), C1s (60.72%) and P2p (3.29%), respectively. At high pH, the content of Fe and Mn decreased, and carbon elements remained unchanged, indicating that iron and manganese oxides participated in the chemical reaction during the adsorption process (Fig. 6d). The result was consistent with the results of EDS spectra of FM-MBC.

**Table 3:** The XPS peak spectral analysis before and after adsorption.

	Bond Energy (eV)					Composition (%)				
	C1s	Fe2p	Mn2p	P2p	O1s	C1s	Fe2p	Mn2p	P2p	O1s
Before	283.92	710.25	710.25	–	529.37	60.72	7.19	3.16	–	26.98
After	284.15	711.13	711.13	133.27	530.96	60.72	5.29	1.30	3.29	27.31

## Conclusion

The study investigated the dynamic adsorption of phosphate ions in aqueous solution using the FM-MBC. Iron and manganese oxides loaded on both the surface and pores of FM-MBC by SEM-EDS, XPS and BET analysis. The removal of phosphate ions from water through fixed-bed dynamic adsorption was influenced by several factors, including solution pH, adsorbent bed height, initial phosphate concentration, and flow rate. Under the optimal condition (initial phosphate concentration = 30 mg L^−1^, pH = 3.0, bed height = 2.0 cm, and flow rate = 3.0 mL min^−1^), the phosphate adsorption capacity was found to be 22.11 mg g^-1^. Furthermore, the solution pH significantly influenced the adsorption capacity of FM-MBC for phosphate ions.

The dynamic adsorption experimental data were fitted with four different models: Thomas, Yoon-Nelson, Adams-Bohart, and BDST. The R^2^ values for the nonlinear regression ranged from 0.9249 to 0.9999, which showed that nonlinear regression is most appropriate for analyzing the dynamic adsorption models. The R^2^ values of the Thomas and Yoon-Nelson models showed slight variation (0.9684 ≤ R^2^ ≤ 0.9963 and 0.9819 ≤ R^2^ ≤ 0.9999), indicating that the dynamic adsorption process of FM-MBC was more complex. Additionally, the study evaluated the regeneration performance of FM-MBC. After four desorption and regeneration cycles, the result suggests that FM-MBC can be effectively regenerated and reused using 0.1 mol L^−1^ NaOH solution. The XRD analysis demonstrated that the reaction product between FM-MBC composite and phosphate anions was Fe_3_(PO_4_)_2_·H_2_O, with the dominant adsorption mechanism being a chemical adsorption reaction. Lastly, the present study proposes that FM-MBC composite is a recyclable adsorbent for removing phosphorus-containing wastewater.

### Supplementary Information

Supplementary Information.

## Data Availability

The datasets supporting the study's findings are available from the corresponding author upon reasonable request.
