# Supplementary material for: Study on the dynamic adsorption and recycling of phosphorus by Fe–Mn oxide/mulberry branch biochar composite adsorbent
Source: Sci Rep. 2024 Jan 12;14:1235. doi: 10.1038/s41598-024-51416-w (PMC10786881; doi:10.1038/s41598-024-51416-w)
Supplement: Supplementary file 1 — Supplementary Information. [file 41598_2024_51416_MOESM1_ESM.docx]

***Supplementary Material for***

**Study on the dynamic adsorption and recycling of phosphorus by Fe-Mn oxide/mulberry branch biochar composite adsorbent**

Liang Meina ^a, b^, Mushi Qiao ^a^, Qing Zhang ^a,^ *, Shuiping Xu ^a^, Dunqiu Wang ^a, b^

*a.* *School of envormental science and engneering, Guilin Unversity of Technology, Guilin 541004, People's Republic of China*

*b.* *Collaborative Innovation Center for Water Pollution Control and Water Safety Guarantee in Karst Area, Guilin 541004, People's Republic of China*

**^*^Corresponding author**

Dr. Qing Zhang, ORCID iD: 0000-0003-1473-0592.

E-mail: [zhangqing@glut.edu.cn](mailto:zhangqing@glut.edu.cn)

Tel: +86773 3693255

S1 Regeneration efficiency

The regeneration efficiency (%) was calculated using the Eqs. (S1):

$Regeneration\text{ }Efficiency=\frac{Q_{r}-Q_{0}}{Q_{0}}\times100\%$ (S1)

Where *Q_r_* represents the adsorption capacity of the regenerated biochar, mg g^-1^; *Q_0_* is the adsorption capacity of fresh biochar, mg g^-1^.

**S2 Adams-Bohart model**

Adams-Bohart established a fundamental equation based on surface reaction theory, which describes the relationship between *C_0_/C_t_* and *t* in a continuous system. The Eqs. (S2) is expressed as follows:

$\frac{C_{t}}{C_{0}}=e^{(K_{AB}C_{0}t-K_{AB}N_{0}\frac{Z}{U_{0}})}$ (S2)

*C_0_* is the initial phosphorus concentration in the influent, mg L^-1^; *C_t_* is the phosphorus concentration in the effluent at time t, mg L^-1^;t is time, minutes; *K_AB_* is the constant of the Adams-Bohart model, L mg^-1^·min^-1^; *N_0_* is the maximum adsorption capacity of the unit volume of the adsorbent bed, mg L^-1^; *H* is the height of the adsorbent bed in the adsorption column, cm; *U_0_* is the empty bed contact time in the column, cm min^-1^.

**S3 Thomas model**

The Thomas model is one of the most commonly used dynamic adsorption models for describing breakthrough curves in fixed-bed systems and predicting the adsorption capacity of adsorbents. It is primarily based on the assumption that there is no axial dispersion during the adsorption process. The adsorption process follows the Freundlich isotherm and pseudo-second-order kinetic reaction. The Eqs. (S3) is expressed as follows:

$\frac{C_{t}}{C_{0}}=\frac{1}{1+e^{(\frac{K_{TH}q_{TH}m}{u}-K_{TH}C_{0}t)}}$ (S3)

*C_t_* is the phosphorus concentration in the effluent at time t, mg L^-1^; *K_TH_* is the Thomas rate constant, mL mg^-1^·min^-1^; *q_TH_* is the saturation adsorption capacity, mg g^-1^; *m* is the mass of the adsorbent, g; *u* is the flow rate, mL min^-1^; *t* represents time, minutes.

**S4 Yoon-Nelson model**

The Yoon-Nelson model is the simplest expression of a classic dynamic adsorption model, which is only suitable for describing the adsorption process of simple component adsorbent systems. The Eqs.(S4) expression is as follows:

$\frac{C_{t}}{C_{0}}=\frac{e^{(K_{YN}t-\tau K_{YN})}}{1+e^{(K_{YN}t-\tau K_{YN})}}$ (S4)

*C_t_* is the effluent concentration at time t, mg L^-1^. *C_0_* is the initial influent concentration, mg L^-1^; *K_YN_* represents the adsorption rate constant, min^-1^; *τ* represents the time required for the solution concentration to reach 50% of the initial concentration, min.

**S5 Bed-depth/service time (BDST) analysis**

Hutchins (1973) introduced the use of the linearised Adams-Bohart model in bed-depth/service time (BDST) analysis to predict the relationship between column bed depth (*Z*) and service time (*t*). BDST analysis allows the prediction of adsorption capacity of continuous adsorption systems. The BDST equation (Eq. (S5)) in the form of *t*=*aZ*-*b* is given by

$t=\frac{N_{0}}{C_{0}U_{0}}Z-\frac{1}{k_{BDST}C_{0}}\ln(\frac{C_{0}}{C_{t}}-1)$ (S5)

Where k_BDST_ (mL mg^-1^ min^-1^) is the kinetic constant, and *a* (slope) and *b* (intercept) are given respectively by:

$a=\frac{N_{0}}{C{}_{0}U_{0}}$ (S7)

$b=\frac{1}{k_{BDST}C_{0}}\ln(\frac{C_{0}}{C_{t}}-1)$ (S8)

The BDST analysis is useful for adsorption process design, where it allows the prediction of the effects of changing flow rates and inlet sevoflurane concentrations without the need to carry out many tests during scale-up. BDST analysis was applied to predict the breakthrough time of the fixed bed systems at 5% and 95% breakthrough.

**S6 Figure caption**

**
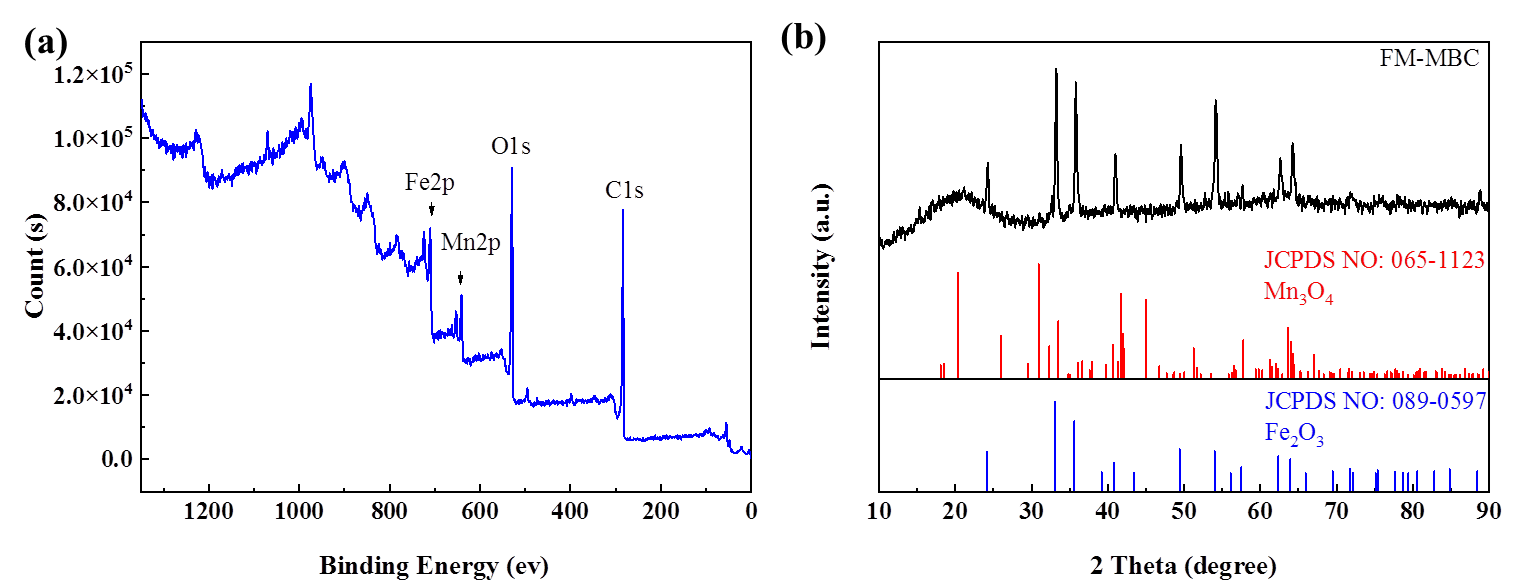
**

**Fig. S1**. The XPS (a) and XRD (b) of FM-MBC.

**Fig. S2.** Zeta potential


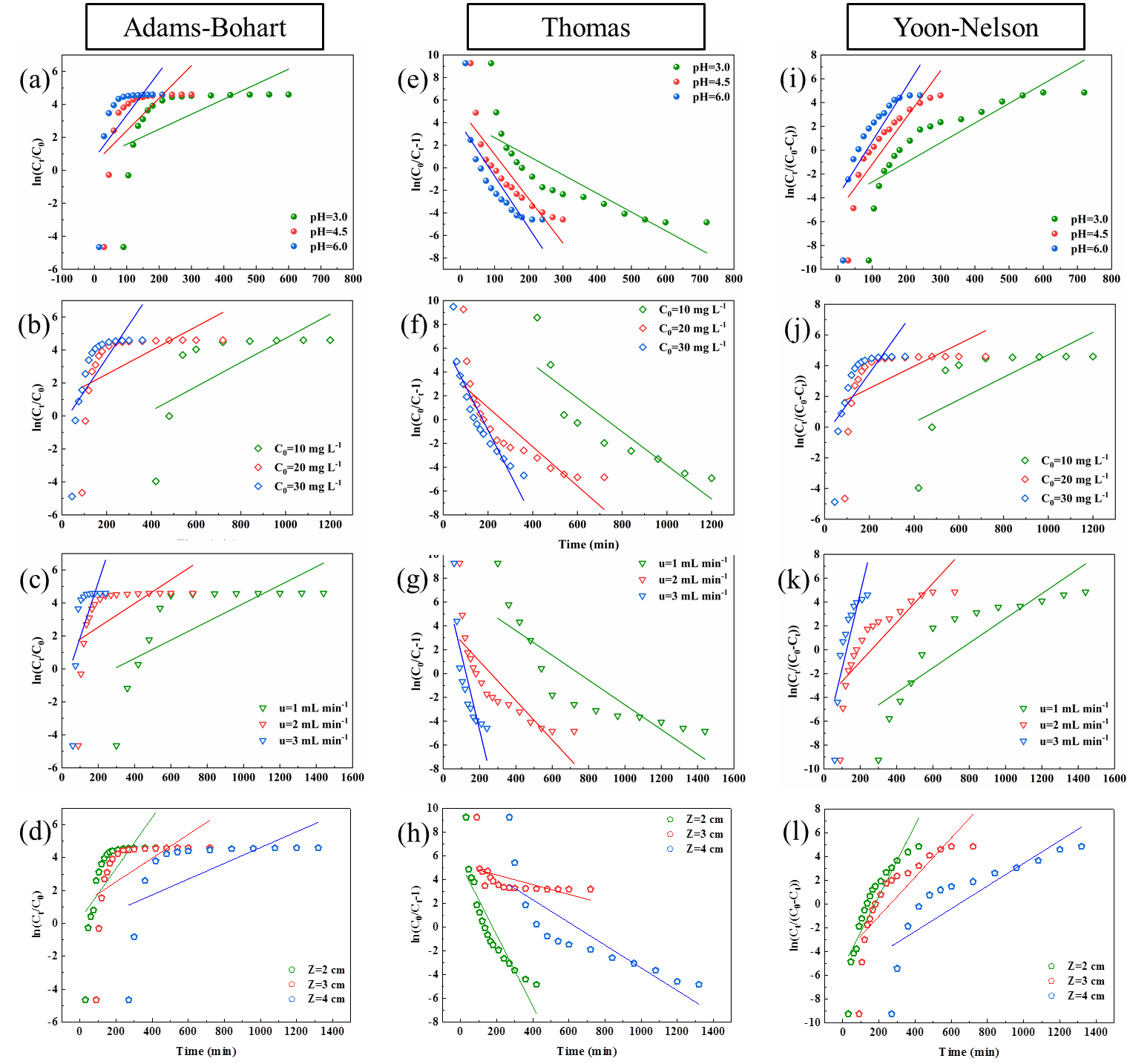


**Fig. S3.** Adams-Bohart, Thomas model and Yoon-Nelson model fitting curves of different solution pH, initial phosphorus concentration, bed height and flow rates (a-d: Adams-Bohart model, e-h: Thomas model, i-l: Yoon-Nelson model)





**Fig. S4.** Analysis of the desorption effect of eluents, NaOH/HCl solution.

**S7 Table**

**Table S1** specific surface area and aperture analysis of FM-MBC

|  | Surface area S_BET_ (m^2^/g) | Pore volume (cm^3^/g) | Average pore size (nm) |
| --- | --- | --- | --- |
| FM-MBC | 318.53 | 0.337 | 4.391 |

.

**Table S2** Parameters of Adams-Bohart, Thomas and Yoon-Nelson and model for dynamic adsorption of phosphate

| C_0_  (mg/L) | u  (mL/min) | Z  (cm) | pH | Adams-Bohart model | | |  | Thomas model | | |  | Yoon-Nelson model | | |
| --- | --- | --- | --- | --- | --- | --- | --- | --- | --- | --- | --- | --- | --- | --- |
|  |  |  |  | K_AB_*10^-3^  (L/mg·min) | N_0_  (mg/L) | R^2^ |  | K_TH_*10^-3^  (mL/mg min) | q_TH_  (mg/g) | R^2^ |  | K_YN_*10^-3^  (min^-1^) | τ  (min) | R^2^ |
| 20 | 2 | 3 | 3.0 | 0.39 | 7972.96 | 0.401 |  | 0.84 | 6524.92 | 0.754 |  | 17.06 | 300.05 | 0.804 |
| 20 | 2 | 3 | 4.5 | 1.02 | 3543.34 | 0.469 |  | 1.97 | 3268.07 | 0.802 |  | 47.80 | 141.79 | 0.794 |
| 20 | 2 | 3 | 6.0 | 1.09 | 2745.09 | 0.372 |  | 2.30 | 2120.40 | 0.712 |  | 45.95 | 83.33 | 0.719 |
| 10 | 2 | 3 | 3.0 | 0.70 | 8097.25 | 0.725 |  | 1.18 | 8634.09 | 0.920 |  | 11.83 | 676.17 | 0.920 |
| 30 | 2 | 3 | 3.0 | 0.70 | 6352.59 | 0.597 |  | 1.22 | 6615.66 | 0.880 |  | 16.25 | 204.89 | 0.621 |
| 20 | 1 | 3 | 3.0 | 0.28 | 9292.73 | 0.642 |  | 0.50 | 19251.03 | 0.833 |  | 10.00 | 724.35 | 0.832 |
| 20 | 3 | 3 | 3.0 | 1.74 | 4439.20 | 0.620 |  | 2.98 | 5200.95 | 0.849 |  | 59.59 | 119.23 | 0.849 |
| 20 | 2 | 2 | 3.0 | 0.79 | 10651.86 | 0.520 |  | 1.49 | 4491.92 | 0.830 |  | 15.85 | 277.89 | 0.520 |
| 20 | 2 | 4 | 3.0 | 0.26 | 12138.64 | 0.511 |  | 0.47 | 15877.20 | 0.810 |  | 5.18 | 955.73 | 0.511 |

C_0_ (mg L^-1^): initial concentration; u (mL min^-1^): flow rate; Z (cm): bed height; K_AB_ (mL min^-1^mg^-1^): Adams-Bohart constant rate; N_0_ (mg L^-1^): saturation concentration of the column; q_TH_ (mg g^-1^): adsorption capacity; K_TH_ (mL mg^-1^ h^-1^): Thomas constant rate; K_YN_ (mL mg^-1^ h^-1^): Yoon-Nelson constant rate.

**Table S3** Parameters of BDST model for adsorption of P on FM-MBC at different conditions.

| C_0_ (mg/L) | Z  (cm) | u  (mL/min) | t_0_._05 actual_  (min) | t_0.05 theory_  (min) | ε_0.05_  (%) | t_0.95 actual_  (min) | t_0.95 theory_  (min) | ε_0.95_  (%) |
| --- | --- | --- | --- | --- | --- | --- | --- | --- |
| 20 | 2 | 2.0 | 60 | 37 | 38.33 | 240 | 184 | 23.33 |
| 20 | 3 | 2.0 | 120 | 167 | 39.17 | 430 | 544 | 26.51 |
| 20 | 4 | 2.0 | 320 | 297 | 7.19 | 960 | 904 | 5.83 |

C_0_ (mg/L): initial concentration; u (mL/min): flow rate; Z (cm): bed height; t_0_._05 actual_ (min): actual breakthrough time (C_t_/C_0_=0.05); t_0.05 theory_ (min)_:_ theoretically calculated breakthrough time (C_t_/C_0_=0.05); t_0_._95 actual_ (min): actual breakthrough time (C_t_/C_0_=0.95); t_0.95 theory_ (min)_:_ theoretically calculated breakthrough time (C_t_/C_0_=0.95).
